# Heregulin Activity Assays for Residual Testing of Cell Therapy Products

**DOI:** 10.1186/s12575-021-00157-5

**Published:** 2021-11-12

**Authors:** Paula V. Monje, Ketty Bacallao, Gabriela I. Aparicio, Anil Lalwani

**Affiliations:** 1 Stark Neurosciences Research Institute, Department of Neurological Surgery, Indiana University School of Medicine, Indianapolis, Indiana USA; 2 Interdisciplinary Stem Cell Institute, University of Miami Miller School of Medicine, Miami, Florida USA; 3 Instituto de Investigaciones Biotecnológicas “Rodolfo A. Ugalde”, Universidad Nacional de San Martín and Consejo Nacional de Investigaciones Científicas y Técnicas (IIBio-UNSAM-CONICET), Buenos Aires, Argentina; 4 Cell and Gene Therapy CMC and Regulatory Advisor, Boulder, CO USA

**Keywords:** Schwann cells, Peripheral nerve, In vitro culture, Autologous cell therapy, Residual testing, Quality control, Signal transduction, ErbB/HER, Kinase activation, Phosphor- antibodies, Western blot

## Abstract

**Background:**

Heregulin is a ligand for the protooncogene product ErbB/HER that acts as  a key mitogenic factor for human Schwann cells (hSCs). Heregulin is required for sustained hSC growth in vitro but must be thoroughly removed before cell collection for transplantation due to potential safety concerns. The goal of this study was to develop simple cell-based assays to assess the effectiveness of heregulin addition to and removal from aliquots of hSC culture medium. These bioassays were based on the capacity of a β1-heregulin peptide to elicit ErbB/HER receptor signaling in adherent ErbB2+/ErbB3+ cells.

**Results:**

Western blotting was used to measure the activity of three different β1-heregulin/ErbB-activated kinases (ErbB3/HER3, ERK/MAPK and Akt/PKB) using phospho-specific antibodies against key activating residues. The duration, dose-dependency and specificity of β1-heregulin-initiated kinase phosphorylation were investigated, and controls were implemented for assay optimization and reproducibility to detect β1-heregulin activity in the nanomolar range. Results from these assays showed that the culture medium from transplantable hSCs elicited no detectable activation of the aforementioned kinases in independent rounds of testing, indicating that the implemented measures can ensure that the final hSC product is devoid of bioactive β1-heregulin molecules prior to transplantation.

**Conclusions:**

These assays may be valuable to detect impurities such as undefined soluble factors or factors for which other biochemical or biological assays are not yet available. Our workflow can be modified as necessary to determine the presence of ErbB/HER, ERK, and Akt activators other than β1-heregulin using native samples, such as fresh isolates from cell- or tissue extracts in addition to culture medium.

## Background

Residual testing of manufacturing process reagents with potential or known adverse effects in humans is an essential quality control step for the preclinical validation of cell therapy products. For quality assurance and compliance with current regulations, the Food and Drug Administration of the USA (USA/FDA) expects validation of the process used for the removal of residuals along with the confirmation that the levels in the final product lie within an acceptable range. Depending on the nature of the residuals, the sponsor is expected to develop, qualify, and eventually validate appropriate methods to demonstrate the removal or reduction of critical product- and process-related impurities [1].

Human Schwann cells (hSCs) obtained via in vitro culture techniques are regarded as valuable therapeutic products to promote regeneration, (re)myelination and neuroprotection [2]. The ability to derive autologous hSC cultures from human peripheral nerves has provided a basis for clinical trials evaluating hSC transplantation for treating central [3, 4] and peripheral nerve trauma [5]. hSCs are most commonly procured from the patient’s sural nerve. However, the number of hSCs isolated from the initial harvest is insufficient for grafting and expansion in culture is required [4]. Successful propagation of isolated hSCs is accomplished only with the addition of serum, usually fetal bovine serum (FBS), and defined chemical factors, including heregulin (an ErbB ligand also known as glial growth factor/GGF or neuregulin/NRG) and forskolin (a cAMP-inducing agent), [4]. Heregulin is the most potent known mitogenic factor for hSCs [6, 7] and is critical for expanding the cell populations through several passages [8].

The discovery that a small fragment of the β1-heregulin protein comprising the EGF (epidermal growth factor)-like domain is sufficient to induce hSC proliferation [6] motivated the use of this peptide rather than the whole protein in media formulations, as this approach is cost-effective to generating large-scale hSC preparations [4]. Heregulin binds to and activates membrane receptors from the ErbB/HER family of receptor tyrosine kinases (RTKs). Several members of the NRG family and the ErbB receptor family, in particular ErbB2, are regarded as protooncogenic factors because they have transforming potential [9]. Thus, strict measures are necessary to guarantee that clinical-grade hSC suspensions are heregulin-free before they are transplanted into humans.

Consequently, the goal of this study was twofold: (1) to design and implement an appropriate test to detect bioactive heregulin in the culture medium; and (2) to confirm the efficacy of the process used to remove soluble impurities from the final, transplantation-grade hSC product. As such, we developed sensitive cell-based assays to detect ErbB- activation to assess the bioactivity of a heregulin-containing sample in live cells. The assays involve the following basic steps: (1) stimulating cultured, adherent, heregulin-responsive, ErbB+ cells with a test sample along with appropriate positive and negative controls for bioactive β1-heregulin, and (2) measuring the expression of ligand-activated ErbB3 (the heregulin-binding component of the ErbB co-receptor), and downstream signaling intermediates (namely, ERK/MAPK and Akt/PKB) by Western blot using phospho-specific antibodies against key activating residues. Data from these assays were interpreted under the assumption that the relative intensities of ErbB3, ERK and Akt phosphorylation were proportional to the concentration of β1-heregulin in a given sample of culture medium. Detection of the soluble β1-heregulin peptide was made possible by biological amplification of ligand-induced ErbB activity in cultures of adult nerve-derived rat SCs. There are several advantages to using rat SCs for mechanistic studies regarding heregulin/ErbB signaling because: (1) they express physiological levels of ErbB2 and ErbB3 mRNA and protein but no other heregulin-binding receptors that elicit transduction through the abovementioned kinase effectors; (2) they exhibit virtually no constitutive, or heregulin-independent activation of ErbB2 and ErbB3; and (3) they readily proliferate in an ErbB-, MEK-ERK and PI3K-Akt-dependent manner when β1-heregulin stimulation is restored [10–12].

Results from these assays confirmed the successful removal of recombinant β1-heregulin peptide from preclinical hSC cultures [13]. In addition, our assays are flexible and can be adapted to monitor other residual factors that impinge on ErbB, ERK and/or Akt activation. In the following sections, we provide in-depth information and technical details relevant to assay development, optimization and use for independent implementation and prospective applications including but not restricted to residual testing.

## Materials and Methods

### Cell Culture Materials, Antibodies, and Reagents

Cell culture-grade water, high-glucose Dulbecco’s Modified Eagle’s medium (DMEM), DMEM/F12 (1:1) with L-glutamine, 15 mM HEPES and without phenol red (Invitrogen, Cat 11039), Hanks Balanced Salt Solution (HBSS, calcium- and magnesium-free), Dulbecco’s Phosphate-Buffered Saline (DPBS), 0.5% trypsin-EDTA, 10,000 U/mL penicillin-streptomycin and 50 mg/mL gentamycin were obtained from ThermoFisher (Waltham, MA). Fetal bovine serum (FBS) was obtained from HyCloneTM-GE Healthcare (Logan, UT) and decomplemented *in-house*. Forskolin, poly-L-lysine (PLL), mouse laminin (1 mg/mL) and phorbol 12-myristate 13-acetate (PMA) were obtained from Sigma-Aldrich (St. Louis, MO). The EGFR/ErbB2 inhibitor 4557 W (CAS 179248–61-4) was from Calbiochem-Novabiochem Corp, La Jolla, CA. The following primary antibodies used for immunostaining were obtained from DAKO (Carpinteria, CA): anti-S100β (rabbit polyclonal, Cat # Z0311) and anti-GFAP (glial fibrillar acidic protein rabbit polyclonal, Cat # Z0334). The following antibodies used for Western blot were obtained from Cell Signaling Technology: anti-phosphorylated (Ser217/221)-MEK (cat. # 9121), and anti-phosphorylated-tyrosine (Cat. # 9411). Alexa Fluor™ 488 and 546-conjugated antibodies in the form of goat anti-rabbit IgG (Cat # A-11035), and goat anti-mouse IgG (Cat # A-11001) were acquired from ThermoFisher. Thy1.1 antibodies were prepared *in-house* from hybridoma cell cultures (ATCC, Manassas, VA), according to our published protocols [14]. All disposable plasticware and cell culture dishes were obtained from Corning. Recombinant human heregulin-β1_177–244_ (HRG1-B1, Preprotech, cat # G-100-03) was prepared in sterile water and stored in aliquots at − 80 °C, as recommended by the manufacturer. Other reagents and equipment are described in the appropriate sections.

### Preparation and Characterization of Stock Cultures of Rat SCs

All procedures involving animals were approved by the University of Miami Animal Care and Use Committee. Rat SC cultures were established from adult (3-month-old) female Fisher rat sciatic nerve tissue, as previously described [11, 12]. Briefly, the sciatic nerve tissue was cut into small segments and allowed to degenerate in vitro by incubation for 2–3 weeks in DMEM medium supplemented with 10% heat-inactivated FBS (DMEM-10% FBS). Degenerated nerve explants were dissociated with a mixture of 0.25% dispase and 0.05% collagenase, and the resulting cell suspensions were plated on PLL-coated 10-cm dishes in DMEM-10% FBS. Contaminating fibroblasts were removed by a complement reaction using Thy 1.1 antibodies. The resulting purified SCs were expanded in DMEM-10% FBS medium supplemented with 2 μM forskolin, 20 μg/ml bovine pituitary extract (Biomedical Tech., Stoughton, MA), and 10 nM β1-heregulin (Genentech). SCs were expanded in the aforementioned medium up to passage-2 before cryogenic storage. Experiments were carried out using cryopreserved cells prepared in RecoveryTM medium or dimethyl sulfoxide (DMSO) and FBS at a ratio of 1:9. Cryopreservation was performed to create large enough stocks of rat SCs from the same batch for use in all heregulin activity assays, thus minimizing lot-to-lot variability in the kinase activation measurements. Adult rat SC cultures consisted of > 98% SCs based on immunostaining with the SC markers S100B or GFAP, and the fibroblast marker Thy1.1 [15]. Cultures obtained by this method were analyzed by RNAseq to confirm the purity and identity of the cells. These cultures expressed ErbB2 and ErbB3 mRNAs but lacked NRG isoforms, EGFR and ErbB4. More information on the RNAseq analysis of rat SC cultures can be found in our prior publication [7].

### Preparation and Storage of in-Process Residual Testing Samples, Positive and Negative Controls

Samples from the culture medium of hSCs were obtained from 3 independent process qualification runs carried out in a facility operating under current Good Manufacturing Practices (cGMP). Several manufacturing process steps and in-process controls were employed to ensure that the final hSC product was free of process-related contaminants [1]. Due to the autologous, per patient-derived nature of the investigational product, fresh organ donor tissue was used for the analysis of β1-heregulin and other process-related residuals such as mouse laminin, gentamicin, and bovine serum albumin [5, 13]. Briefly, connective tissue-free endoneurial fascicles from donor sural nerves were cultured for one week in β1-heregulin- and forskolin-supplemented medium. The predegenerated fibers were dissociated with collagenase and neutral protease to produce a cell suspension that was plated at low density in laminin-coated culture flasks for propagation. When the hSC cultures were ~70% confluent, the cells were enzymatically dissociated and re-plated for another round of subculture under identical conditions to generate pure, transplantation-grade hSCs. These cells were detached and rinsed three times using large volumes of serum-free DMEM/F12 medium before final resuspension in transplantation solution. Test samples for heregulin activity assays were collected from the hSC-conditioned medium (culture supernatant from the final hSC cultures) and from the washing steps identically during each process qualification run. The supernatants from rinses 2 and 3 were recovered for in-process testing prior to collection of the cell pellet (transplantable product). These samples were snap-frozen on dry ice and stored at − 80 °C until testing was performed.

The conditions tested in the assays were as follows: (1) DMEM/F12 with no additives (negative control); (2) freshly prepared hSC growth medium containing 10% FBS, 10 nM β1-heregulin peptide and 2 μM forskolin both provided from a stock solution (positive control); (3) growth medium diluted 10, 100, 1000, 10,000, and 100,000 times in DMEM/F12 (serial dilutions for estimation of dose-dependent changes); (4) Medium from semi-confluent hSC cultures before trypsinization (i.e., hSC conditioned medium), (5) 2nd and 3rd wash supernatants from hSC suspensions prepared as per transplantation protocols (test articles); and (6) 3rd wash supernatant spiked with 1:10 and 1:100 of fresh growth medium. Spiked samples were tested only in 2 out of 3 process qualification runs to control for potential heregulin-modifying elements in the final wash medium. All samples used to stimulate rat SCs were prepared in advance and subjected to one freeze-thaw cycle for consistency. Of note, the ErbB3-, ERK- and Akt-inducing activities were not notably different between frozen and fresh samples of the same culture medium (data not shown).

### Plating, Starvation and Stimulation of Rat SCs for Heregulin Activity Assays

Cryovials of rat SCs were quickly thawed at 37 °C and re-suspended in 10–15 mL DMEM containing 10% FBS before collection by centrifugation. The cells were then plated directly onto PLL-coated 10-cm dishes at a density of 1–2 × 10^6^ cells/dish in high-glucose DMEM, 10% FBS, 10 nM heregulin, 2 μM forskolin, and antibiotics [16]. Cells were cultured in a 37 °C humidified incubator set to 9% CO_2_ until they were 60–80% confluent (usually after 4–5 days) with regular media changes every 2–3 days. To obtain a single cell suspension, the cells were dislodged from the dishes via enzymatic treatment with trypsin/EDTA prepared in calcium- and magnesium-free HBSS. The cells were then plated into test dishes (24-well) coated with PLL and mouse laminin to promote strong adhesion of the rat SCs particularly during the starvation period. The rat SCs were seeded at a density of 100,000 cells per well in 0.5 mL DMEM containing 10% FBS (high serum) and immediately transferred to a CO_2_ incubator. Twenty-four hours later, the medium was replaced with an equivalent volume of HEPES-buffered DMEM containing 1% FBS (low serum), and the cells were  incubated for an additional 24-h period in a CO_2_ incubator. Sequential deprivation of mitogens and serum was required to prevent massive apoptotic cell death, as adult rat SCs are sensitive to abrupt changes in medium composition and pH, particularly after the removal of mitogens and serum [17]. Traces of serum and mitogenic factors were removed on the day of experimentation by replacing the medium with pre-warmed, serum-free DMEM/F12 (500 μl/well) followed by stabilization for ~ 1 h in a CO_2_ incubator. DMEM/F12 was chosen to both maintain the lowest possible endogenous kinase activity pre-stimulation and prevent pH changes. To initiate the assay, the medium was replaced expeditiously with 500 μl of the pre-warmed (37 °C) samples from the control and test articles, as described above, and the cells were transferred immediately to a CO_2_ incubator for the times indicated in the figures. To terminate the assays, the medium was removed by aspiration and the cells were lysed as described below. Each experimental condition was analyzed in duplicate samples with similar Western blot signals.

 Further technical details on rat SC culturing and coating of 24-well dishes can be found in our previous publications [14, 16]. Briefly, a 0.02% PLL working solution was used to completely cover the surface of the wells for 1 h at room temperature (RT) before incubation for at least 1 h with a laminin solution prepared at a ratio of 0.4–0.6 μg laminin per cm^2^ of coated surface. The laminin solution was removed immediately before seeding the cells to ensure a fast adhesion and even distribution of the rat SCs as soon as 15–30 min post-plating. For consistency of results, we used passage 3–4 rat SCs from the same stock in all experiments.

### Protein Lysate Preparation, Electrophoresis and Western Blot

Total cell lysates were prepared as previously described [12, 18, 19] with minor modifications. To expedite the cell lysis and maintain the phosphorylation signal as stable as possible at the time of sample collection, the culture medium was rapidly removed using a glass pipette connected to a vacuum line and the cells were lysed directly in the wells by the addition of 150 μl pre-warmed (50 °C) SDS-sample buffer (225 mM Tris/HCl, pH 6.8, 5% SDS, 50% glycerol, 250 mM dithiothreitol, 0.5 mg/ml bromophenol blue) for electrophoresis under denaturing conditions (SDS-PAGE). Protein lysates were transferred to 1.5 mL Eppendorf tubes expeditiously and denatured by boiling for 10 min in a heating block at 100 °C. Protein aliquots were resolved in denaturing (SDS) 10% polyacrylamide gels (1 mm thick, 15-lane gels) using15 μl of sample per lane. A colored molecular weight marker (Fermentas, Cat. # SM1811) was loaded in the first and last lanes of each gel as a reference for the consistent electrophoretic separation and transfer of the proteins. Electrophoresis was performed at a constant voltage of 80 V and stopped once the bromophenol blue dye reached the bottom of the gel. The average electrophoresis time under these conditions was 1.5–2 h. Fractionated proteins in the gels were transferred to polyvinylidene fluoride membranes (Immobilon-P membranes, Millipore, Bedford, MA Cat. # TM 151–1) using a standard liquid transfer protocol and a transfer time of ~ 2–3 h. Membranes were blocked for 30 min in Tris-buffer saline (TBS, Bio-Rad) containing 0.05% Tween-20 (TBS-T) and 2% ECL blocking agent (ECL Blocking Reagent, GE Healthcare Life Sciences, Cat. # RPN2125). The membranes were incubated with antibodies diluted in ECL blocking solution overnight (~ 20 h) at 4 °C, with continuous shaking. Next, the membranes were rinsed 3x with TBS-T and incubated with a 1:10,000 dilution of horseradish peroxidase-conjugated secondary antibodies (Goat anti-mouse IgG-HRP, Cat. # sc-2055, and goat anti-rabbit IgG-HRP, Cat. # sc-2301, Santa Cruz Biotechnology) in ECL blocking solution for 1 h at RT. The membranes were washed 3 times with TBS-T and immunoreactive proteins were detected by enhanced chemiluminescence (ECL) using ECL Plus detection reagents (GE Healthcare Life Sciences, Cat. # RPN2132) according to the manufacturer’s instructions. Light-sensitive film (Amersham Hyperfilm ECL, GE Healthcare Life Sciences, Cat. # 28–9068-39) was exposed according to the signal intensity of each blot, typically necessitating 1 to 30 min exposure time.

The electrophoresis and transfer equipment used was as follows: Mini PROTEAN II electrophoresis cell (Biorad, Cat # 165–2940), Mini Trans-Blot module (Bio-Rad, Cat. # 170–3935); and PowerPac Basic Power Supply (Bio-Rad, Cat. # 164–5050). The development of ECL membranes was performed using Automatic Film Processor, Konica Minolta, Model # SRX-101A. Gels were prepared in-house using 30% acrylamide / bis-acrylamide solution, 29:1, (Bio-Rad, Cat. # 1610156) and sodium dodecyl sulfate (SDS, Bio-Rad, Cat. #1610301). The electrophoresis, transfer and incubation buffers were prepared using the following reagents: 10x Tris/Glycine/SDS (Bio-Rad, Cat. # 161–0772); 10x Tris/Glycine buffer (Bio-Rad, Cat. # 161–0771); 10X TBS (Bio-Rad, Cat. # 1706435); Tween20 (Sigma, Cat. # P9416); Glycerol (Sigma, Cat. # G5516); 1,4 dithiothreitol (Roche, Cat. # 708984), bromophenol blue sodium salt (Sigma, Cat. # B8026). The antibodies used in heregulin activity assays were the following: pAkt-Ser-473 (Santa Cruz, Cat # sc7985, rabbit polyclonal, 1: 500); pERK1/2/MAPK (Santa Cruz, Cat # sc7383, mouse monoclonal, 1:500); pErbB3-Tyr-1289 (Cell Signaling, Cat # 4791S, rabbit monoclonal, 1:1000); Akt (Cell Signaling, Cat #. 9272, rabbit polyclonal, 1:1000); ERK2/MAPK (Santa Cruz, Cat # sc154, rabbit polyclonal, 1:500), and ErbB3 (Santa Cruz, cat # sc285, Rabbit polyclonal, 1:500). Other antibodies listed in the Materials section were used at 1:500.

### Densitometric Analysis of Western Blot Bands

The relative optic density (O.D.) for each band was estimated using the gel analysis tool in ImageJ (NIH) available at (https://imagej.nih.gov/ij/). Scanned Western blot images (300 dpi) were transformed into grayscale mode and the background was digitally subtracted before analysis. The regions of interest were defined for each scan in sequential order, starting with the negative controls. The area under the curve method was used to quantify the intensity of the bands and to generate profile plots in which the height of the peaks represents the density of the bands in the original membranes. All quantified proteins were represented by discrete, single bands except for the 44/42 kDa doublet detected by P-ERK and total ERK antibodies. The 44/42 kDa doublets were enclosed within the same rectangular region of interest and quantified together for practical reasons. Data are represented as arbitrary O.D. units respective to the negative control value, or as the ratio between the phosphorylated and total protein bands for each protein species to obtain the relative level of phosphorylation at each condition. Images with uneven or interrupted bands were not used for densitometric quantification.

### Cell Proliferation Assays

The incorporation of the thymidine analog BrdU into nuclear DNA was assayed as a measure of S-phase entry, as previously described [12]. Briefly, adult rat SCs were plated on PLL/laminin-coated 24-well dishes (50,000–70,000 cells/well) in DMEM supplemented with 10% FBS. One day later, the medium was changed to HEPES-buffered DMEM containing 1% FBS (non-proliferating medium), to induce quiescence. The cells were stimulated with mitogenic factors in the absence or presence of 10 μM of 4557 W (referred to as ErbB2 inhibitor or ErbB2i) for 3 days in medium containing BrdU (1 μM). Quiescent rat SCs typically incorporate BrdU within 24–48 hs after heregulin stimulation. To detect incorporated BrdU, the cells were fixed sequentially with 4% paraformaldehyde and − 20 °C methanol, and blocked with 5% normal goat serum. Cells were treated for 30 min with a 50% solution of DNase in the presence of anti-BrdU (1:300) and then incubated with Alexa594-conjugated secondary antibodies. BrdU staining was performed alone or together with SC markers.

### Immunofluorescence Microscopy

The cells were double fixed with paraformaldehyde and methanol, as described above, and blocked with 5% normal goat serum in PBS before the addition of antibodies against P-ERK (1:200) or GFAP (1:300) and incubation overnight at 4 °C. The cells were washed with PBS, incubated with fluorescent secondary antibodies (1:300) prepared in 5% normal goat serum containing the nuclear stain DAPI, and mounted for microscopy using Vectashield. Images were taken using a cooled digital CCD camera (SensiCam QE, Cooke Corp.) coupled to an Olympus IX70 inverted fluorescence microscope. Black and white images (8-bit, tiff format) were artificially colorized in RGB format, digitally processed and arranged for presentation using Adobe Photoshop 21.0.2 and Adobe Illustrator 24.0.1.

### Statistical Analysis

Statistical comparisons were performed using GraphPad Prism software, Version 4 (GraphPad Software, Inc., San Diego, CA, USA). Experimental data are expressed as the mean ± standard error of the mean (SEM) of samples from 3 independent experiments. One–way ANOVA was used followed by Bonferroni’s post hoc test. The results were considered significant when * *p* < 0.05.

## Results

### Designing a Workflow for the Testing of Residual Heregulin-Associated Activity in Culture Medium Samples

FDA guidance for cellular and gene products suggests screening for residual manufacturing reagents with known or potential adverse effects [1]. Since the manufacture of hSCs uses a recombinant β1-heregulin peptide (referred to as β1-heregulin or heregulin in the text), laminin, trypsin, collagenase, and neutral proteases, among other components, testing was required to detect those soluble components in the final hSC product. Ensuring the removal of β1-heregulin was crucial because of its potential tumorigenic effects. Although extensive rinsing of the cell suspensions results in the dilution of the β1-heregulin activity by several orders of magnitude, it was conceivable that traces of this mitogen could remain in the final hSC product or could be released by the cells themselves during processing, potentially leading to undesirable effects in patients.

The analysis of residual heregulin was challenging due to the lack of suitable tests for detecting the small β1-heregulin peptide used to supplement culture media, which consists of a 65 amino acid, 7.5 kDa molecule containing the EGF-like domain of β1-heregulin. Typically, a 10 nM concentration of this mitogenic β1-heregulin peptide is provided in FBS- and forskolin- supplemented medium both for the culturing the nerve fascicles (pre-degeneration step) and expanding the hSCs through serial passaging [6, 8]. We found that Western blot analysis was insufficient to detect nanomolar concentrations of the abovementioned polypeptide diluted in culture medium (data not shown). Therefore, we decided to take advantage of the high affinity of its binding to ErbB3 receptors, and the possibility of measuring the activation of kinase effectors via immunological methods [12], to detect ligand (heregulin)-dependent, ErbB-induced activity in living cells. As such, we developed simple assays based on biological amplification of heregulin-initiated signaling, referred to as ‘Heregulin Activity Assays’, to determine the presence of heregulin-associated bioactivity in unprocessed samples of hSC culture medium.

**Fig. 1:**
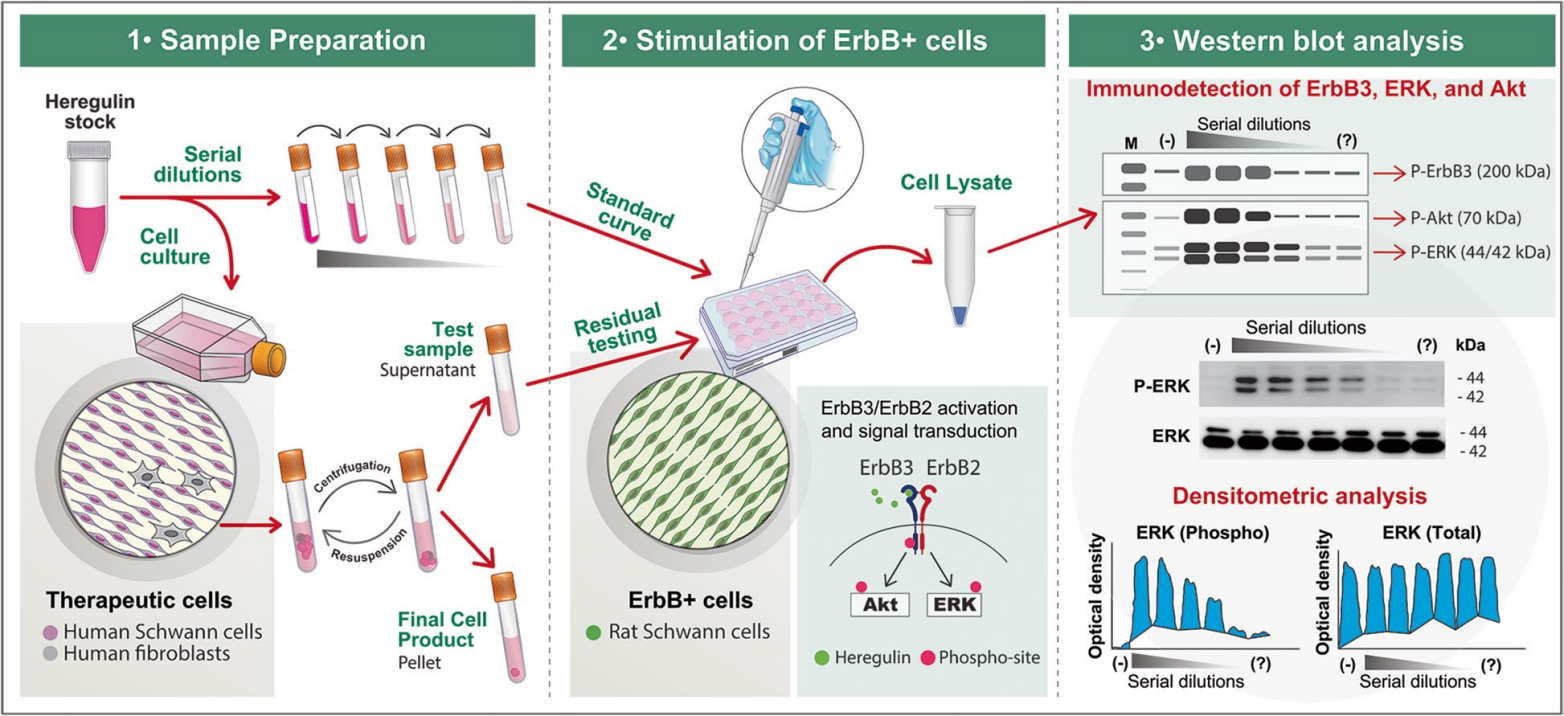
Residual testing of heregulin in the manufacture of hSCs for transplantation. The diagram outlines the workflow used to test heregulin activity directly using aliquots of culture medium (panel 1). In these assays, mitogen- and serum-starved rat SCs (panel 2) were used for their capacity to effectively bind and transduce signals from heregulin-activated ErbB2/3 (inset in panel 2). Detection was carried out using antibodies against the total and phosphorylated forms of ErbB3, ERK and Akt followed by incubation with HRP-conjugated secondary antibodies. Immunoreactive bands were visualized by capturing the product from an ECL reaction on light-sensitive film. Quantification of the Western blot data was done by densitometric analysis using ImageJ. The total operating time from onset (plating of the rat SCs) to completion (data analysis) was 4–5 days on average. An example of P-ERK and T-ERK detection and quantification from a typical gel is shown in panel 3. The ERK signal is revealed as a doublet consisting of closely related isoforms ERK1 and ERK2 (panel 3)

The schematic diagram presented in Fig. 1 summarizes the overall workflow for the assays consisting of the following basic steps: (1) stimulating mitogen and serum-deprived rat SCs with the supernatant (or wash media) of transplantable hSCs along with positive and negative controls; (2) preparing cell lysates, running an electrophoresis, and performing a Western blot using phospho-specific antibodies against total (reference controls) and phosphorylated kinase substrates (readout signals); and (3) using densitometric analysis to quantify the intensity of the phosphorylation bands in test samples in comparison with those in the positive and negative controls.

**Fig. 2:**
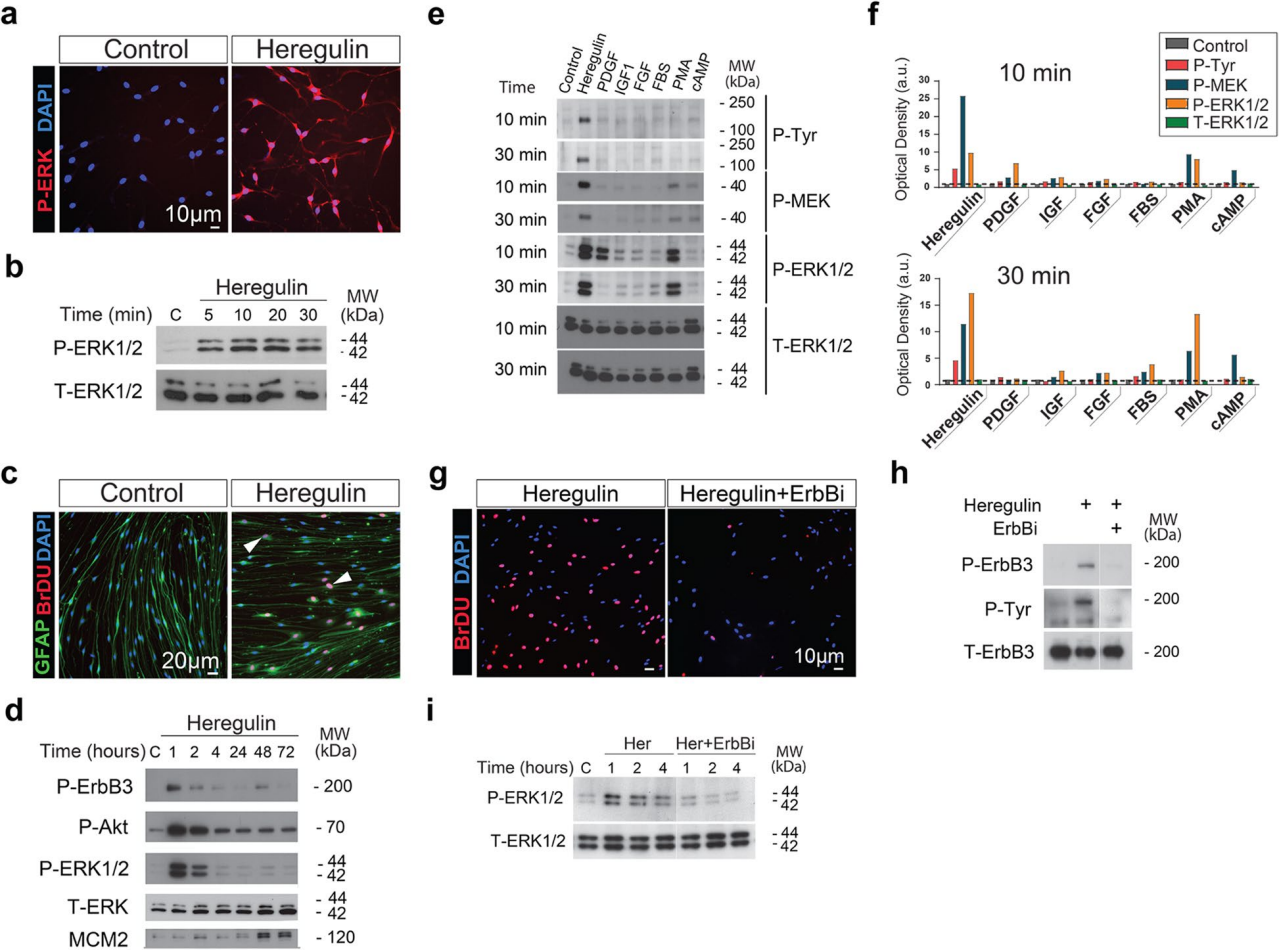
Characterization of heregulin-induced kinase activation in rat SCs. **a-b** Heregulin-induction of ERK phosphorylation in adult nerve-derived rat SCs as evidenced by immunofluorescence microscopy (**a**, 10 min post-stimulation) and Western blot (**b**, as indicated). **c-d** Heregulin-elicited rat SC proliferation as evidenced by BrdU incorporation (**c**) and induction of MCM2 expression (Western blot, **d**). Note the delay between the onset of MCM2 expression and the peaks of ErbB3, ERK and Akt phosphorylation (**d**). **e,f** Activity and specificity of RTK-induced signaling in response to assorted growth factors and serum at two early time points, as indicated. Note that β1-heregulin elicits the most potent and long-lasting activating signal when compared to other growth factors, serum and cAMP-stimulating agents. **g-i** Effect of a pharmacological ErbB inhibitor (ErbBi) on heregulin-induced SC proliferation (BrdU incorporation, **g**), ErbB3 tyrosine phosphorylation (**h)** and ERK activation (**i**). An antibody against total P-tyrosine (P-Tyr) was used to reveal generic changes associated with RTK activation in rat SCs (**e-f** and **h**). The phorbol ester PMA was used as a positive control for receptor-independent, PKC-mediated MEK and ERK phosphorylation (**e,f**). In panel **d**, the time course of expression of MCM2 is shown to confirm the expected β1-heregulin-associated changes in cell cycle progression under the same conditions used for kinase stimulation in the same batch of rat SCs. The β1-heregulin peptide was provided at 10 nM in all experiments

As explained in the following sections, the optimization of the assays required the preparation of a validated batch of rat SC cultures, the selection of the most appropriate kinase readouts and stimulation times, and a determination of the assay’s sensitivity to render reliable data in independent experimental rounds. Western blot was the preferred detection method because it is a standard and highly sensitive technique to assess the phosphorylation of assorted substrates. Three kinase readouts were selected for Western blot experiments, namely ErbB3, Akt and ERK, based on their ample activation range and heregulin-dependence in rat SCs [12]. ErbB3 (~ 200 kDa), Akt (~ 70 kDa) and ERK1/2 (~ 44/42 kDa) are resolved very clearly by SDS-PAGE and can be detected on the same Western blot membrane using validated, highly selective total and phospho-specific antibody combinations that do not cross-react with other antigens. Expanded adult rat SCs (normal, established from nerve-derived primary cells) were chosen for the stimulation experiments because consistent responses to soluble β1-heregulin were expected [12, 19, 20]. Adult rat SCs constitutively express ErbB2 and ErbB3, which dimerize to form a functional heregulin co-receptor (Fig. 1), and are highly responsive to β1-heregulin with the induction of proliferation (Fig. 2c), [11, 12, 20]. The responses of adult rat SCs to β1-heregulin are rapid, potent and specific to ErbB2/3 activation (Fig. 2). Signal specificity in SCs is optimal because: (1) ErbB3 is the only ligand binding partner of heregulin-like molecules, and (2) ErbB2 does not display constitutive activation/phosphorylation in the absence of the ligand [10–12, 19, 20]. In fact, the phosphorylation of kinase effectors downstream of ErbB2-ErbB3 can be lowered to negligible levels by removing the ligand from the culture medium (Fig. 2a-b, d-e), which is an advantage for mechanistic studies of kinase pathways. In addition, the intensity and duration of ErbB2-ErbB3 activation can be enhanced with pharmacological management of the intracellular levels of cAMP without altering the specificity or heregulin-dependency of ErbB activity [10].

### Preparation and Validation of ErbB+ Cells Responsive to β1-Heregulin Peptide

The main advantage of using cultures of rat SCs is that they are phenotypically stable and can be amplified substantially through serial passaging without becoming heregulin-independent [21]. By expanding SCs from adult rat nerves, it was feasible to prepare an excess of cells (> 10^10^ cells per batch) from a single well-characterized stock and store them by cryopreservation for use in all optimization and validation rounds essentially as if they were a cell line. For consistency, the batch of rat SCs used for the heregulin activity assays was investigated deeply for its responsiveness to β1-heregulin (Fig. 2a-e), the expression of phenotypic markers (shown for GFAP, Fig. 2c), and the lack of fibroblast contamination (as per Thy1.1 immunodetection, not shown). The identity of the cells was further examined by means of high throughput gene expression profiling (RNAseq), [7]. RNAseq data indicated a > 17-fold difference in the expression levels of ErbB3 and ErbB2 transcripts, whereas the expression levels of EGFR, ErbB4, EGF and NRG isoforms were negligible. The expression levels of selected transcripts (in RCPMs) were the following: Erbb2 (37.739 ± 1.141); Erbb3 (635.663 ± 27.444); Erbb4 (0.350 ± 0.0748); Egfr (0.139 ± 0.047); Nrg1 (0.253 ± 0.109); Nrg2 (0.450 ± 0.094); Nrg3 (0.106 ± 0); Nrg4 (0.146 ± 0.0355); Egf (0.335 ± 0.052). More information on the gene expression profiling of this batch of rat SCs can be found in the NCBI Gene Expression Omnibus (GEO) database (accession number GSE133716 and ID 200133716).

### Optimization of Stimulation Conditions and Detection of Heregulin-Dependent Kinase Targets

**Fig. 3:**
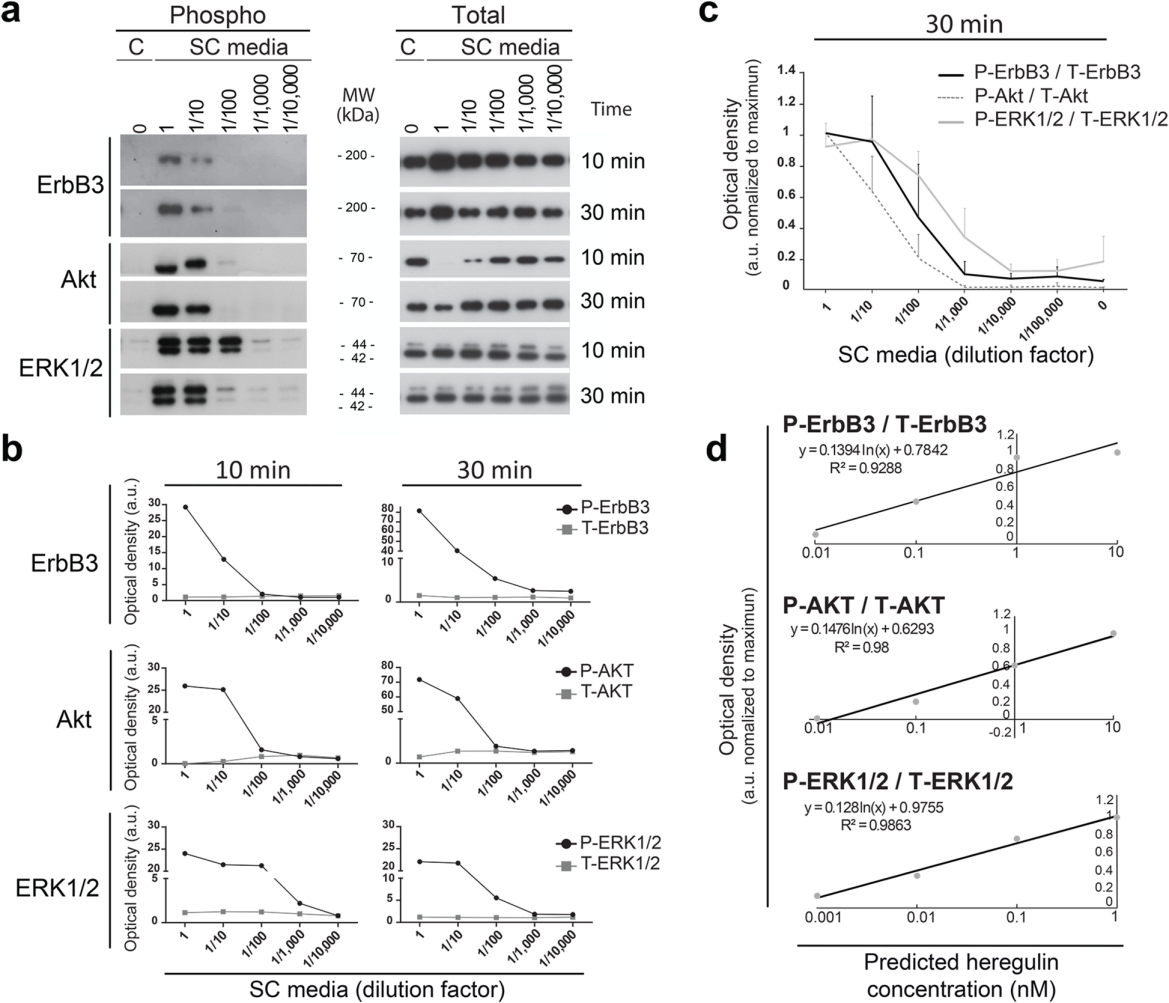
Dose-dependent activation of ErbB3, ERK and Akt in response to stimulation with β1-heregulin-supplemented medium. **a,b** Freshly-prepared hSC culture medium was diluted up to 1:10,000 in DMEM/F12 and used to stimulate rat SCs for 10 and 30 min, as indicated, with nearly identical results. SC growth medium was used as a positive control (maximal stimulation) and DMEM/F12 was used as a negative control (first lane). The antibodies selected for these experiments specifically recognized the phosphorylated forms of ErbB3, ERK1/2, and Akt on Tyr-1289, Tyr-204, and Ser-473, respectively (**a**). Antibodies against ErbB3, ERK1/2 and Akt were used for a reference to total levels of expression regardless of the phosphorylation state of these kinases. Phospho- and total-proteins were quantified by densitometric analysis, normalized to the negative control condition, and expressed as arbitrary units of O.D. for each condition at each time point (**b**). The scale of the y axis (panel **b**) was partitioned to evidence that the changing levels in total protein do not explain the changes in each respective phospho-protein. **c,d** Comparison of dose-dependency curves for P-ErbB3, P-Akt and P-ERK (**c**) and detection range for each kinase (**d**) using densitometric data from 3 independent experiments. Regression analysis detailing the sensitivity range for each kinase to resolve changes in heregulin concentration **(d)** based on data shown in **(c).** Experiments were conducted as explained in **a,b** with the exception that a fixed time point (30 min) was used. Densitometic data were normalized to the value of the maximum signal to establish a relevant comparison among data from the different readouts. All measures allowed detection of heregulin in the range of 10–0.01 nM but no signal resolution as a function of heregulin concentration was possible > 1 nM (for ERK) or 10 nM (for ErbB3 and Akt). Samples containing < 0.001 nM heregulin were indistinguishable from heregulin-free samples

The activity of the above described β1-heregulin peptide, and other ligands from the NRG family, can be measured by assaying ErbB-dependent pathway activation in rat and human SCs [10–12, 19]. One advantage of SCs is that they readily reduce the activation state of virtually all elements of the ErbB pathway upon the removal of β1-heregulin, forskolin and FBS from the culture medium. Thus, a critical step in setting up the conditions for stimulation was to design a starvation protocol to sequentially remove serum and mitogenic factors without altering the adhesion or the survival of the SCs. It was evident that rat SCs became quiescent within 2-to-3 days after the onset of starvation, as evidenced by the lack of BrdU incorporation into nuclear DNA in starved cells (Fig. 2c, control). These changes occurred together with the downregulation of ErbB, MEK-ERK and Akt phosphorylation to often undetectable levels, as judged by detection with phospho-specific antibodies in immunofluorescence microscopy (Fig. 2a, shown only for ERK) and Western blot experiments (Fig. 2b-d). Yet, SCs readily activated ErbB receptor signaling (and in turn entered the S-phase of the cell cycle) when nanomolar doses of β1-heregulin were provided alone (Fig. 2a-e) or in conjunction with other standard media components (Fig. 3).

Heregulin-induced ErbB, ERK and Akt activation was transient, and a drastic decline was observable at 1–2 h post-stimulation. This effect was particularly noticeable in the activation kinetics of ErbB3 and ERK1/2, which both returned to basal levels unless other factors were added to the medium (Fig. 2d), [11]. Whereas Akt phosphorylation was maintained for a longer period, the intensity of the signal was reduced at 1–2-h post-induction (Fig. 2d). High and fairly stable phosphorylation signals for all kinases were evident for up to 30 min before a decay was noticeable under the conditions of our assays.

Therefore, we decided to investigate the activity and specificity of the β1-heregulin-elicited phospho-signals at 2 early time points post-stimulation, 10 and 30 min. As shown in Fig. 2e-f, β1-heregulin-induced kinase phosphorylation was specific when assessed at these time points (shown in the activation of ERK and its upstream kinase, MEK). Other polypeptide growth factors, serum (FBS), and cAMP-elevating agents did not drive substantial tyrosine phosphorylation (P-Tyr) or MEK-ERK activation. PDGF only transiently induced the phosphorylation of MEK and ERK (Fig. 2e). The ErbB-dependency of β1-heregulin-induced ERK phosphorylation (Fig. 2i) was confirmed using a selective ErbB/EGFR pharmacological inhibitor that effectively bocks SC proliferation and ErbB3 tyrosine phosphorylation in rat SCs (Fig. 2g-h).

Altogether, these results confirmed the time-dependency, specificity and potency of the β1-heregulin-induced activation of ErbB3, ERK and Akt as determined by Western blot. These three kinases displayed the highest signal resolution in the Western blot experiments, with consistent and reproducible results. Other substrates, including ErbB2 [12], MEK [12], RSK, GSK3 [20], CREB, and PKC, were confirmed to be valid heregulin-responsive targets in rat SCs but the activation kinetics and dose-dependency in response to the β1-heregulin peptide were not characterized in-depth due to the lower resolution or limited range of activation of the phosphorylation signals (data not shown).

### Time and Dose-Dependency of Culture Media-Elicited Kinase Activation

Next, we investigated the potency of serial dilutions of β1-heregulin-supplemented hSC culture medium to construct reference curves and ascertain the dose-dependency of the ErbB3, ERK and Akt phosphorylation signals. Our first analysis compared responses at 2 early time points (i.e., 10- and 30-min post-stimulation) as the signal intensity reflecting the phosphorylation state of all kinases decays dramatically with longer incubation times (Fig. 2d). Results presented in Fig. 3a-b suggested that the P-ERK signal provided the highest dose sensitivity likely due to intracellular amplification through the Raf-MEK signaling axis. However, the phospho-Akt signal was more stable and the difference in intensity was the highest to the control condition, an advantage for signal resolution by ECL detection. Although the P-ErbB3 signal was highly specific and clearly demonstrated dose-dependent changes, it showed the weakest response, and efficient detection of its phosphorylation on film usually required longer exposure times.

Thus, to more accurately evaluate the consistency of our method to detect heregulin bioactivity across a range of concentrations, we analyzed samples from cells that were stimulated at a single time point. We chose a 30 min stimulation time for these experiments because this time frame was both informative and practical for the concomitant analysis of a larger number of samples in independent rounds. The results from our comparative quantitative analysis (Fig. 3c-d) indicated a non-overlapping dose-dependent activation of the 3 kinases. Under our selected experimental conditions, signal discrimination was effective at doses ranging from 10 to 0.01 nM heregulin for ErbB3 and Akt, and 1 to 0.001 nM for ERK (Fig. 3d, regression analysis). The detection of heregulin-elicited phospho-signals at this range of concentrations was expected from the low dissociation constant for heregulin-ErbB3 binding [22, 23]. Our method did not resolve differences in heregulin concentration at doses > 10 nM nor detected bioactivity in samples that contained < 0.001 nM heregulin, regardless of the readout used.

**Fig. 4:**
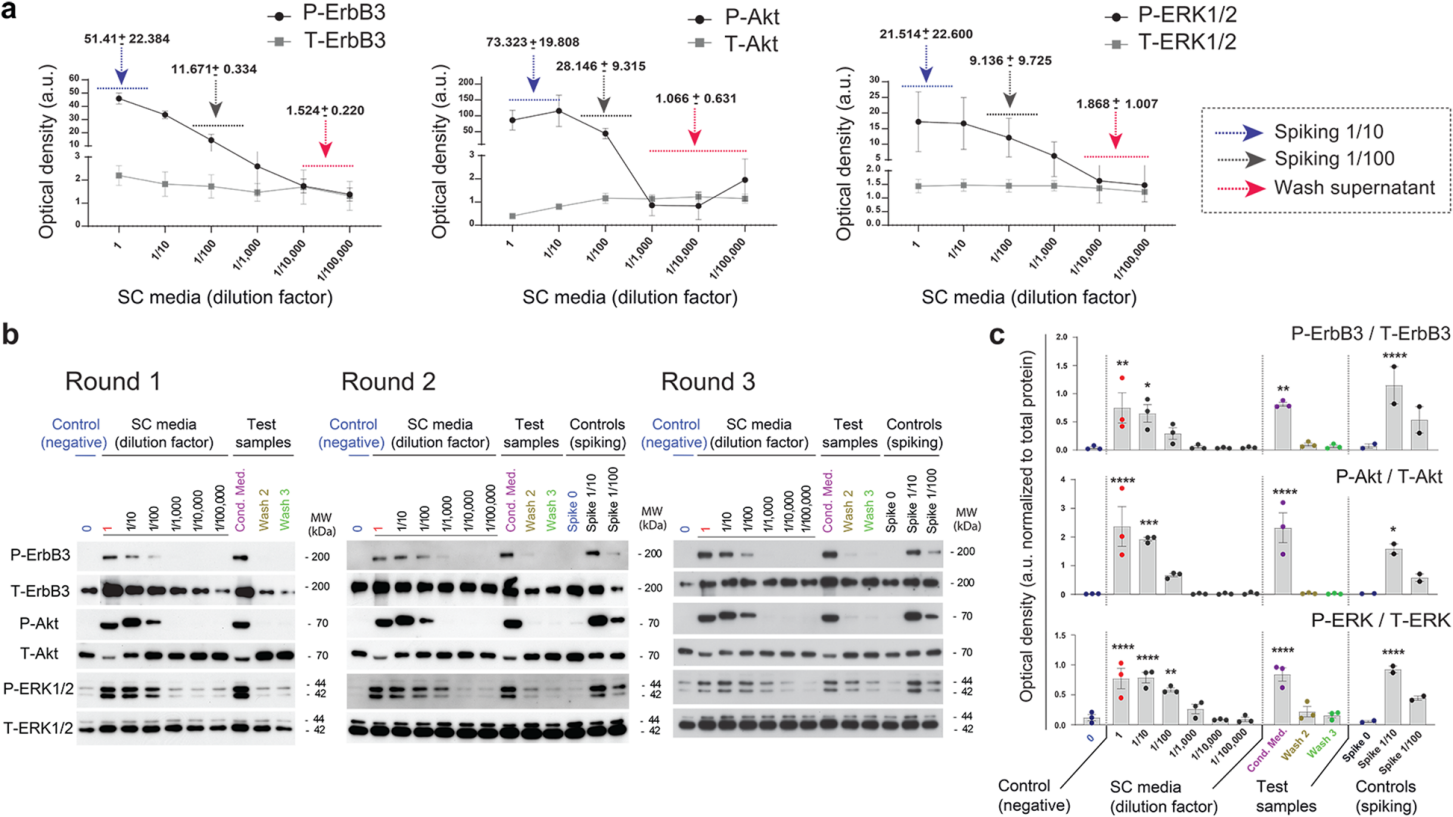
Validation rounds of dose-dependent changes elicited by fresh hSC media, conditioned media, and wash media derived thereof. Experiments were conducted and analyzed as described in Fig. 3 using a 30 min time for stimulation. **a** Dose-dependency curves for culture medium samples highlighting the expected concentration ranges for positive controls (spiking samples) and final wash media (test samples). **b-c** Original Western blot data (**a**) and densitometric quantification from 3 rounds of testing (**c**). The test samples, the negative, and the positive controls were snap-frozen on dry ice and stored at − 80 °C up until stimulation of starved rat SCs. The samples consisted of the following: DMEM/F12 (negative control), serial dilutions (up to 1:100,000) of SC growth medium (standard curve), conditioned medium (i.e., cell culture supernatant exposed to cells) and spiking controls at 1/10 and 1/100 (positive controls). Samples for each experimental condition were obtained and analyzed by Western blot in duplicate. Because duplicate samples rendered nearly identical activation profiles, data from one representative membrane per experimental condition is shown. The spiking controls (depicted in the 2 last lanes from each gel) were included for rounds − 2 and − 3 only. To aid in interpretation, the Western blot profiles were aligned horizontally and the blots from each phospho-antibody (readout signal) were located above the respective total protein, (**b**). In (**c**), data from each phospho-kinase were normalized to the respective total levels of protein and expressed as arbitrary units of O.D. Values of significant comparisons between the negative control condition and the culture medium conditions or the test articles conditions were indicated in the graphs according to their degree of significance (* *p* < 0.05, * * *p* < 0.01 and * * * *p* < 0.001). Note that heregulin concentrations in the wash supernatants were not high enough to trigger the activation of ErbB3, ERK or Akt above basal levels. The absence of biological activity in these samples provided a high degree of assurance that the final hSC formulation was free of soluble process residuals

Western blot experiments consistently showed that the total protein levels and electrophoretic mobility of ErbB3 and Akt were temporarily changed in response to activation for reasons that we cannot explain fully. This is not an unexpected finding because post-translational modifications such as phosphorylation often change the stability and molecular weight of membrane receptors and other signal transduction molecules. Nevertheless, it was evident that the changes in phosphorylated proteins did not result from changes in total protein levels (Figs. 3b and 4a). To account for possible differences in total protein expression levels, data were also represented as the ratio between the phosphorylated and total signals in Figs. 3d and 4c.

Collectively, our analyses confirmed that our method could effectively reveal the presence of heregulin at concentrations able to elicit an effect in live cells. The method is not strictly quantitative because of the non-linear output of intracellular, kinase-mediated amplification of heregulin-ErbB signaling but it serves as a first approach to estimate the presence or absence of heregulin bioactivity within a range of concentrations. Importantly, our method rendered consistent responses using a normal (non-transformed) cellular system expressing physiological levels of ErbB receptors.

### Testing of Residual β1-Heregulin in Preclinical hSC Cultures

The final step was to test the presence of process-related β1-heregulin activity using samples of culture medium from independent preparations of hSCs obtained following strict cGMP guidelines. These experiments were carried out using same-lot materials, reagents and cell cultures, as well as identical protocols, to ensure consist kinase phosphorylation levels. As shown in Fig. 4, the data obtained from 3 independent experimental rounds using the supernatants from different batches of cultured hSCs (identified as rounds-1 to − 3 in the figure) yielded nearly identical information. Critically, the activation of ErbB3, ERK or Akt was negligible (validation round 3) or not detected (validation rounds 1 and 2) using medium samples collected from the washing steps, indicating that the heregulin remnants in transplanted cells were under the range of detection. These experiments introduced several positive controls, including conditioned medium from hSC cultures sampled prior to trypsinization and spiking controls of culture medium diluted in wash supernatant, to rule out the presence of heregulin-interfering activity in the wash medium itself (Fig. 4a-c).

We conclude from these experiments that one or more sequential washes of the hSC suspensions were sufficient to remove physiologically active heregulin molecules and undefined (possibly mitogenic) serum or autocrine factors from the conditioned media. Taken as a whole, these data lend confidence to the effectiveness of the steps used to remove soluble components from transplantable hSCs.

## Discussion

### Activity Assays for the Testing of Residual Heregulin

Standard methods for the growth and expansion of hSCs inevitably render a manipulated cell product. Two or more passages using complex media formulations are needed to obtain large enough yields of purified cells for implantation [4]. In turn, various quality control steps are required for any given batch of autologous hSCs to meet release criteria. Testing of manufacturing residuals in hSCs was particularly challenging due to the assorted factors used during tissue dissociation and culturing. Heregulin was a critical residual component for which a specific and effective test was not available when the experiments were performed. This motivated us to develop simple assays *in-house* to test process validation samples from organ donor-derived, transplantation-grade hSC cultures collected from a local cell manufacturing facility. It is pertinent to mention that the data from our assays were deemed appropriate by the FDA to justify the absence of heregulin in autologous hSC preparations and more generally, to confirm the effectiveness of the protocol used for the elimination of other potentially contaminating soluble components [3], considering that the culture medium samples were tested without being altered before measurement.

### Development and Optimization of Experimental Conditions, Controls and Readouts

The first step in assay optimization was to establish the most appropriate conditions and controls to obtain reproducible data in subsequent independent rounds. For this, we determined the time course of activation and the stability of the phosphorylation signal for the different kinase readouts over a range of agonist concentrations. To increase the detection range and maximize the signal-to-noise ratio in the Western blot data, we adjusted a stepwise starvation protocol whereby the rat SCs were deprived of potential kinase agonists prior to stimulation. This step was essential to reduce kinase phosphorylation levels to a minimum at the onset of experimentation. Other variables that required adjustment included the conditions for cell plating and the expedited collection of samples for analysis of phosphorylated proteins. Because of the similarities in the activation kinetics, dose- dependency and specificity of ErbB3, ERK and Akt activation, we conclude that these measures are roughly equivalent, and heregulin-selective, under the conditions of our experiments. Using the results from 3 distinct kinases lends confidence to the reliability of the method while broadening the potential applications of our assays.

It is worth noting that Akt and ERK can be activated by a variety of RTK-dependent and -independent pathways that use these ubiquitous kinases as signal transducers. Rat SCs do not exhibit ligand-independent ErbB2 activation; thus, the tyrosine phosphorylation of ErbB3 is strictly dependent on extracellular (experimentally added) heregulin. Indeed, ErbB3 phosphorylation is the most specific readout for heregulin activity because it directly binds to, and in turn gets activated by, the ligand. One can argue that ErbB3 phosphorylation at Tyr-1289 is likely mediated by ErbB2 because, (1) ErbB3 is a kinase-dead receptor and can only be trans-phosphorylated on tyrosine residues by dimerization with ErbB2 [23]; and (2) rat SCs do not express any other known ErbB co-receptor [6]. The main reason for our choice of ErbB3 rather than ErbB2 as readout was based on the feasibility to detect and resolve changes in its phosphorylation by Western blot. The levels of activated and total ErbB2 in rat SCs are much lower and more variable than those of ErbB3 [12].

### Selection of ErbB+ Cells for Stimulation Experiments

Rat SCs were an ideal cellular system to investigate the activation of the ErbB receptors because ErbB2/3 activation in these cells is almost exclusively dependent on the provision of exogenous ligands. Rat SCs express physiological levels of ErbB2 and ErbB3 but lack the expression of the other family members (EGFR/ErbB1 and ErbB4), and do not normally secrete NRG, which would otherwise lead to autocrine activation of the receptors. Not only are rat SCs highly sensitive to soluble and membrane-bound NRG ligands but the signals from activated ErbB3, ERK and Akt are rapidly downregulated upon ligand deprivation. In fact, an important internal control was to ensure that our rat SC stocks were essentially devoid of fibroblasts, as these contaminating nonglial cells are known to express EGFR and potentially contribute soluble neuregulin-1 protein [24]. Appropriate negative controls were included in all of the experiments to show that the phosphorylation levels of ErbB3, ERK and Akt were negligible in the absence of heregulin.

Although our set-up utilizes rat SCs, other ErbB+ primary cells or cell lines may be validated and used with potentially similar results. Expanded hSC cultures can be considered because, similar to rat SCs, they express high levels of ErbB2 and ErbB3 in the absence of EGFR and ErbB4 [6, 11]. However, hSCs are not recommended because the cultures are more variable (lot-to-lot) and often contain contaminating fibroblasts [7], thus making them a less stable and less consistent cellular system to quantitatively compare fast-changing phosphorylation profiles in independent experiments. In addition, hSCs get senescent with passaging, which is undesirable for independent or larger scale experimentation [6, 7].

### Advantages and Limitations of our Assays

We should emphasize that these assays are flexible and can be adapted accordingly to assess the presence of ligands/factors other than heregulin, and signaling pathways other than ErbB, ERK and Akt. In particular, the phosphorylation of the latter kinases can serve as a readout for undefined serum factors or autocrine molecules released by the cultured cells themselves, since hSCs and fibroblasts express the transcripts of a wide variety of ligands for membrane receptors that use ERK and/or Akt as signal transducers [25]. Though the heregulin activity in the hSC medium is expected to be derived from the recombinant heregulin peptide used as an additive during culture, the contribution of endogenous activity cannot be ruled out, as the hSC cultures themselves can synthesize NRG-related molecules. It has been established that fibroblasts in hSC populations express the transcript for NRG1 [25], thus raising the possibility that some of the heregulin activity detected in hSC-conditioned medium originates from fibroblasts which are highly proliferative and usually populate these cultures in various proportions. It should be noted that our assays cannot discriminate the effect of experimentally added heregulin and heregulin secreted by cells in culture, and/or other factors in the conditioned medium.

We understand that our method can complement rather than replace the information obtained from ELISA assays due to the ability to examine the potency of an ErbB-inducing activity in a native sample. Immunoreactivity for heregulin/NRG (e.g., as determined by an ELISA) may or may not correlate with evoked bioactivity in live cells (our protocol). Our assays are semi-quantitative and appropriate to estimate the presence of ErbB-stimulating signals of biological significance still, within a limited range of concentrations. Though this method was useful for residual testing, it has obvious limitations for accurate dose determinations and detection outside the nanomolar range. The relationship between the phosphorylation of ErbB3 and the phosphorylation of ERK and Akt supports the validity of the results. However, changes in heregulin concentration do not lead to proportional changes in Akt and ERK phosphorylation.

Increasing the number of samples per experiment is feasible considering that rat SCs are not usually limiting for setting up large-scale cell cultures. Here, we have used mini-gels because it was both feasible and practical for our particular need. Yet, the simultaneous analysis of large numbers of samples may be technically challenging because of limitations in the electrophoresis and Western blot steps. Although Western blot and immunofluorescence microscopy are the most popular methods to detect the phosphorylation of these kinases, other methods are becoming available. Scaling to a higher number of samples may be best accomplished using rapid detection systems such as dual ELISA kits for P-ErbB3/ErbB3, P-ERK/ERK and P-Akt/Akt.

### Alternative Applications

Prospective applications of the methodologies described in this report include screening of novel mitogenic, oncogenic or survival factors expressed by (and exerting an action on) SCs. ERK and Akt are ubiquitous transducers of RTKs, G-protein-coupled receptors (GPCRs), adhesion receptors, cytokine receptors, and other extracellular signals. Alternatively, the assays can be used to uncover the existence of novel bioactive molecules, as shown previously in the identification of soluble and membrane-bound ERK/Akt-inducing factors in cultured DRG neurons [19]. It is worth noting that these assays do not replace biochemical assessments via quantitative methods with increased sensitivity such as radioligand binding assays or ELISAs. Even though we were able to expand the detection range by biological amplification of heregulin-initiated signaling, consistent detection of heregulin activity below the nanomolar range seems unlikely under the conditions of these experiments. As such, these assays may be advantageous in preliminary investigations to inform on the possible existence of > 10^− 10^ M concentrations of heregulin-like bioactivity in complex or undefined samples such as tissue or cell extracts without subjecting the samples to chemical or physical modifications, or purification, before testing.

## Conclusions

To summarize, our data confirmed that the phosphorylation/activation of ErbB3, along with that of ERK and Akt, can be used as an accurate readout for the presence of biologically active heregulin in a test sample used to stimulate adherent ErbB+ cells. We have provided exhaustive technical information on assay set up and optimization for independent follow-up along with an example on how to use the assays in preclinical investigations of cell therapy products. We have also explained the rationale for each step and argued that the parameters may be changed for using other test samples, pathway-specific readouts or detection methods. Finally, these assays are easy to implement in laboratory settings equipped with a standard cell culture facility and Western blot equipment. Applications could range from gathering proof-of-concept data on the existence of undefined factors in native samples or examining mechanistic features of known molecules with potential neuroactive properties or those with a yet-to-be-explored mechanism of action.

## Data Availability

Not applicable.

## Abbreviations

Akt: Akt kinase
A.U.: Arbitrary units
cAMP: cyclic adenosine monophosphate
EGF: Epidermal growth factor
ErbB/HER: ErbB/HER receptor tyrosine kinase
ERK: Extracellular signal regulated kinase
DMEM: Dulbecco’s Modified Eagle’s medium
DMSO: Dimethyl sulfoxide
FBS: Fetal bovine serum
FDA: Food and Drug Administration
GPCRs: G protein-coupled receptors
HBSS: Hanks Balanced Salt Solution
hSCs: Human Schwann cells
IND: Investigational New Drug
NRG: Neuregulin
SCs: Schwann cells
RTK: Receptor tyrosine kinase
WB: Western blot
O.D.: Optical density
ECL: Enhanced chemiluminescence
